# Biodeterioration Risk Assessment in Libraries by Airborne Fungal Spores

**DOI:** 10.3390/jof10100680

**Published:** 2024-09-29

**Authors:** Yiniva Camargo-Caicedo, Hilary Borja Pérez, Maryann Muñoz Fuentes, Eliana Vergara-Vásquez, Andrés M. Vélez-Pereira

**Affiliations:** 1 Programa de Ingeniería Ambiental y Sanitaria, Facultad de Ingeniería, Universidad del Magdalena, Santa Marta 470004, Colombia; ycamargo@unimagdalena.edu.co; 2 Grupo de Investigación en Modelación de Sistemas Ambientales-GIMSA, Facultad de Ingeniería, Universidad del Magdalena, Santa Marta 470004, Colombia; 3 Departamento de Ingeniería Mecánica, Facultad de Ingeniería, Universidad de Tarapacá, Arica 1000000, Chile

**Keywords:** *Aspergillus*, book collection, book deterioration, quality of indoor environments, risk assessment

## Abstract

Fungal growth on cellulose-based materials in libraries can have detrimental effects on books and documents. This biodeterioration affects their physical, chemical, and esthetical characteristics. Thus, this work aimed to assess fungal aerosols’ concentrations and biodeterioration risk in two public libraries with artificial ventilation: the Banco de la República and CAJAMAG libraries. Air sampling was performed using a two-stage viable Andersen cascade impactor with Sabouraud dextrose agar at 4% on Petri dishes. Also, the temperature and relative humidity were measured with a digital thermo-hygrometer HOBO U12 Data Logger. The concentrations were low, with values of around 35 CFU/m^3^. *Aspergillus*, *Cladosporium*, and *Penicillium* were the predominant genera in the two libraries, with *A. niger* being the most abundant species. The thermo-hygrometric conditions inside the libraries were stable, with a mean temperature of 25.2 °C and a mean relative humidity of 52.1%. The calculated potential risk associated with fungal aerosols was seen to be medium in both libraries, where *Aspergillus* genera reported the highest cellulosic activity and hence had a higher risk of biodeterioration.

## 1. Introduction

Libraries and archives have been used for centuries to preserve various materials and store valuable information [1]. These documents which can have organic or synthetic compositions do deteriorate over time [2]. This is because these materials are potential nutrients [3] for microorganisms. Several factors can cause risks to materials and people’s health in libraries [4]. Biodeterioration is one of the main factors affecting long-term storage [5], where a complex phenomenon occurs along with other decomposition causes [6]. A prevention protocol by Polo et al. [7] requires the identification of microorganisms as a priority, particularly for both bacteria and fungi [8,9,10].

Fungi, unlike bacteria, are considered the most important organisms as biodeterioration agents of organic matter [11]. Likewise, they have been noted as the most frequent deterioration agents in historical libraries and archives [12]. Among the genera of fungi, the most frequently characterized biodeterioration agents are *Aspergillus*, *Penicillium*, *Cladosporium*, *Chaetomium*, *Fusarium*, *Trichoderma*, *Alternaria*, and *Paecilomyces*. These genera overlap with the airborne fungal spores in the library documented by Camargo-Caicedo et al. [13], where the most common ones are *Aspergillus*, *Cladosporium*, and *Penicillium*. Thus, airborne fungal spores’ concentration in libraries can indicate their presence in the collections, so it is possible to use them as an indirect indicator of the risk of deterioration [13,14].

Fungi causes chromatic alterations via the formation of stains of different colors, shades, and textures due to mycelial growth and pigment production. When they grow on paper, they degrade all the carbon-containing components, such as cellulose, and excrete organic acids deposited on the substrate [14,15,16]. Fungi produce different enzymes, such as cellulase, amylase, xylanase, and gelatinase; some of them are extracellular enzymes, which provoke the breakdown of paper components and could be accelerated by the growth of fungal hyphae [17,18]. The biodeterioration of books can be esthetic, color change [19], or reddish-brown spots [20], but also causes effects like paper weakness [21]. *Aspergillus niger* is the most common species reported with a high potential for the biodeterioration of paper books, showing a higher growth rate on cellulose substrate [22,23,24].

Additionally, airborne fungal spores in libraries are linked with health risks. Most of the previous genera mentioned, or their metabolites/proteins, have been reported as aeroallergens [25,26,27,28,29,30]. This could irritate the respiratory system, affecting the health of workers and visitors [31]. Such effects range from a mild allergy [32] to critical cases of aspergillosis or phaeohyphomycosis [33]. The main route of exposure is contact with the contaminated material [34]. It should be clarified that fungal infections can occur in any age group, but the symptoms differ according to the immunity status of the individual [35]. Fungi may behave as allergens, and if the concentration of spores is over 2.000 UFC/m^3^, they can be seen to elevate the risk factors for health [36].

The fungi grow at 63–100% relative humidity and between a 15 and 35 °C temperature [17]. This growth rate is higher when the temperature is above 23 °C and the relative humidity exceeds 65% [37]. When favorable microclimatic conditions are present, microorganisms are likely to infect library collections and initiate the process of their biodeterioration [38]; these actively growing organisms can form biofilms on surfaces in indoor environments and release spores into the air, dominating the site-specific microbiological community [7]. In addition to high temperature and humidity [1,14,39], other physical and chemical factors, such as dust [14,34] and poor air circulation [37], together with poor ventilation and inhomogeneity in the surface temperature of materials, can produce local water condensation points with a higher availability for fungi or bacteria than in the rest of an indoor environment [7].

Several studies have analyzed the microclimate within historic libraries, but comparisons are difficult because of the lack of long-term microclimatic observations and uniformity in the use of standards and risk assessment methods [40,41]. The methods for assessing the risk from biological agents include BIOGAVAL (Generalitat Valenciana), Osakidetza (Basque Country), classification by groups [42], and the standard operating procedure—PNO [43], among others. However, this methodology is focused on biological occupational risk. Currently, no methodology has been proposed for the biodeterioration of books.

In indoor environments, the biological component of air constitutes a potential element of degradation. Hence, the prompt and effective identification of biological aerosols and the thermo-climatic condition will also be relevant to assessing the collections’ risk conditions [14]. In this sense, this is the first work at the Banco de la República y CAJAMAG libraries in the District of Santa Marta, Colombia, that aimed to assess airborne fungal spores’ concentration and associated biodeterioration risk, identifying the genera and environmental conditions in two public libraries.

## 2. Materials and Methods

### 2.1. Study Area

**Figure 1:**
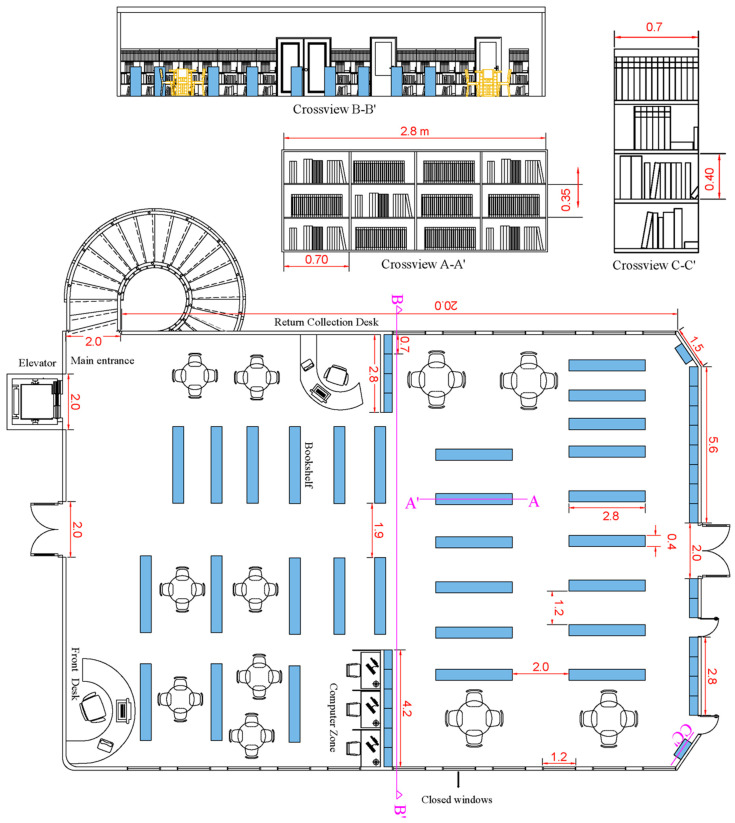
Diagram of the Banco de la República library and detail on the bookshelf.

**Figure 2:**
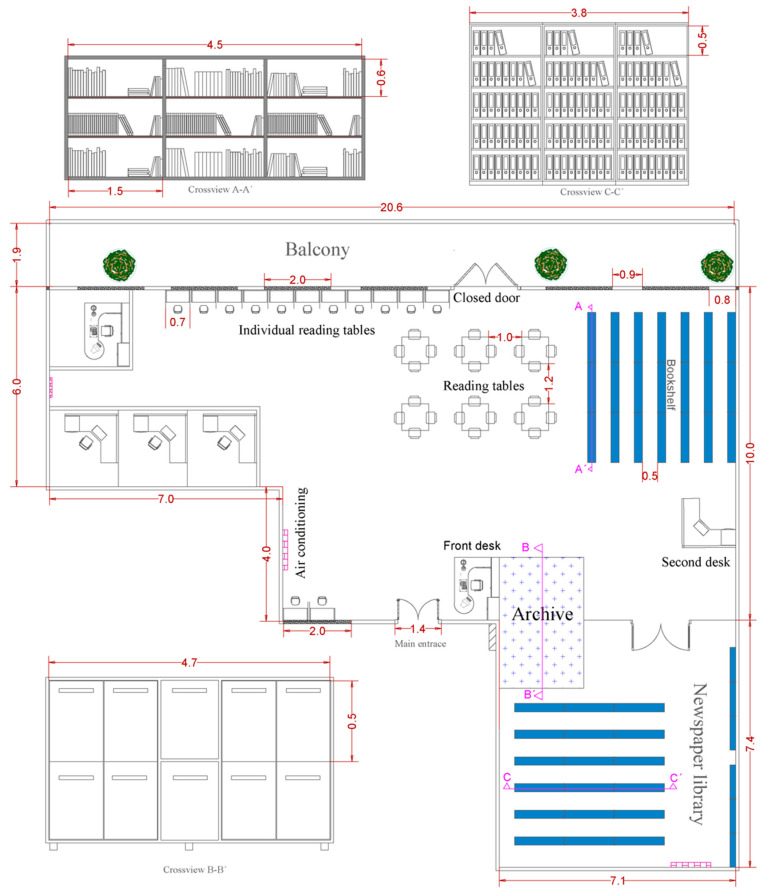
Diagram of the CAJAMAG library and detail on the bookshelf.

This study was undertaken in two public libraries in the District of Santa Marta, Colombia: the Banco de la República and CAJAMAG libraries. The Banco de la República library has an area of 415.8 m^2^, with a bibliographic collection of more than 30,000 copies, and it has a general room made up of 45 open shelves with free access to the collection. It also has a reading room for newspapers and magazines (details in Figure 1). The CAJAMAG library has an area of 233.7 m^2^ and is furnished with seven open shelves, eight closed shelves serving as a newspaper library, and one closed archive area (details in Figure 2). These libraries have artificial ventilation systems by means of air conditioning devices distributed throughout the total available area.

### 2.2. Sampling

The air sampling was performed in five campaigns using a two-stage viable Andersen cascade impactor Model TE-10-860 (Tisch Environmental, village of Cleves, South Miami, OH, USA) for the collection of sedimentable viable particles (stage 1) and respirable viable particles (stage 6). The sample was collected into Petri dishes utilizing 4% Sabouraud dextrose agar (SDA) as a selective culture medium for isolating fungi (Figure S1). The equipment was positioned at a height of 1.5 m, and a flow rate of 28.3 l/min throughout the sampling was used, verified by an air rotameter connected to the pumping system of the impactor. The sample time was determined by preliminary sampling at three time intervals (4, 6, and 10 min) to determine the most efficient sampling practices. These intervals were based on the criteria indicated in previous studies [44,45,46,47]. The optimal time selected was four minutes according to the confidence limits and precision test results. Two monitoring points per library were established: Point 1 in the general room and Point 2 in the reading room. In both cases, the selection criteria used were the location of the collections and the influence of the mass air circulation of the air-conditioning systems. The samples were collected in duplicate (an original and a replica), and each monitoring session was carried out over 40-min intervals in the morning and afternoon. During the sampling inside the libraries, a digital thermo-hygrometer HOBO U12 Data Logger was used to measure the temperature (T, °C) and relative humidity (RH, %).

To ensure the quality of the measurements made in this study, a control protocol was applied for four processes associated with the preliminary steps, during and after monitoring: (1) agar preparation, (2) sampling dishes conditioning, (3) sterilization of the impactor between changing dishes using alcohol and thermal shock (flaming), and (4) control of samples’ incubation conditions (see Figure S2). Equally, other strategies were applied to guarantee the quality of the samples, such as the preparation of additional Petri dishes which were incubated at the end of each monitoring (control dishes) to verify the absence of growth of microorganisms. Similarly, another strategy involved sealing the dishes with paraffin film and wrapping them in plastic film to avoid the entry of particles, microorganisms, or any other external agent.

### 2.3. Sample Processing and Analysis

After the collection of the samples, the processing of these was carried out by incubation of the agar dishes for five days at 25 °C. Once the incubation was completed, the fungal colonies in the Petri dishes were counted to calculate the concentration expressed in colony-forming units per m^3^ (CFU m^−3^). This calculation was realized from the sum of the arithmetic average between the original and replica samples of each stage.

Fungal genera were identified through both macroscopic and microscopic analyses. In macroscopic identification, we register information about color, texture, and edge, among others; in the microscopic examination, each colony was transferred to slides, stained with lactophenol blue, and observed under 10X, 40X, and 100X objectives. The identification was based on fungal morphological characteristics (conidia, spores, and other resistance structures), following the taxonomic keys of Raper and Fennell [48] (1965), Barnett and Hunter [49], and Carrillo [50]. Genera were identified by strictly adhering to these morphological keys and corroborating the identifications using the MycoBank online database (https://www.mycobank.org/ accessed on 20 September 2024). This process was overseen by mycologist Leda Sotomayor (RIP).

### 2.4. Biodeterioration Risk Assessment

A simple methodology has been proposed to assess the potential biodeterioration risk on collections from airborne fungal spore concentrations. The risk calculation is based on the apparition frequency, concentration threshold, and potential biodeterioration of each genus/species. All these criteria were transformed into four categories: low (1), medium (2), high (4), and very high (5). The frequency and concentration thresholds will be established with quartiles from the dataset. In contrast, the potential of biodeterioration was established according to the literature, taking two criteria: (a) if the genus/species is recognized to degrade cellulose and (b) information about their degradation kinetic (if available). The equation to establish the risk is the sum of frequency and concentration thresholds multiplied by biodegradation potential, so the risk scale is between 2 and 50. A value between 2 and14 is low risk, 15 and 26 is medium risk, 27 and 38 is high risk, and 39 and 50 is very high risk.

### 2.5. Statistical Analysis

The samples from each library were analyzed separately, applying one-factor analysis of variance (ANOVA) to determine statistically significant differences between the fungal spore concentrations and each thermo-hygrometric condition (temperature or relative humidity). A multivariate regression was applied to determine the influence of the thermo-hygrometric conditions on the fungal spore concentrations, for which different models were evaluated to establish the best fit. The tests were conducted with a 95% interval confidence and a *p*-value of 0.05 using the statistical software Statgraphics version XVIII.

## 3. Results

### 3.1. Airborne Fungal Spores’ Concentration

**Figure 3:**
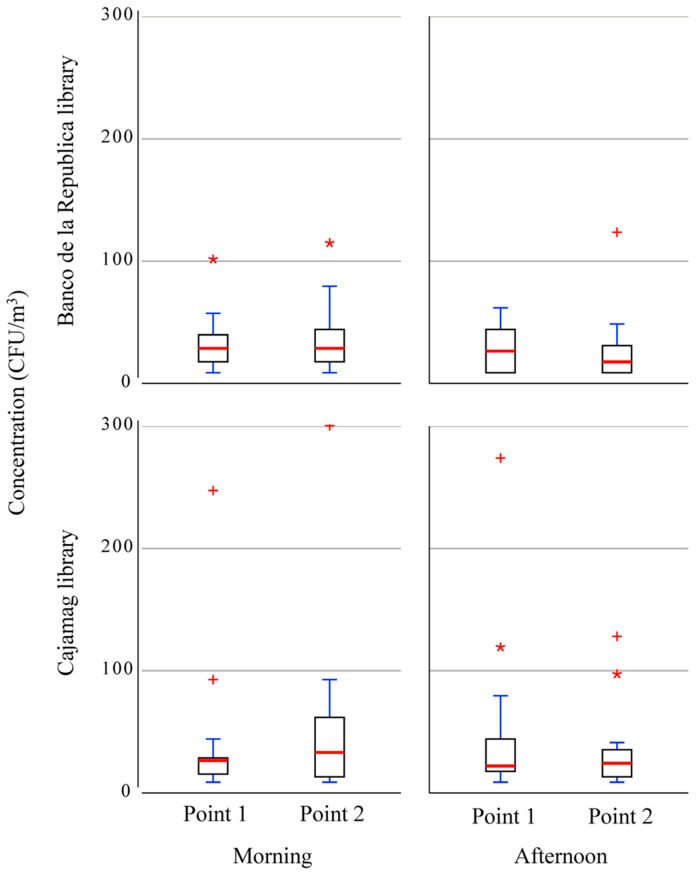
The concentration of airborne fungal spores by library, the time of the day, and sampling point. * Extreme value is 1.5 times more than the 75th percentile. ^+^ Extreme value is three times more than the 75th percentile.

Figure 3 shows the concentration of the fungal aerosols in the libraries under study. Higher maximum concentrations were seen in the CAJAMAG library in comparison with the Banco de la República library with maximum values of 300.4 CFU/m^3^, followed by 273.9 CFU/m^3^ and 247.3 CFU/m^3^, as noted during the morning period. This trend was also seen in the average concentrations, with the CAJAMAG library mean (35.7 CFU/m^3^) concentration being greater than that of the Banco de la República library (25.0 CFU/m^3^), but this difference was less stark than that seen for the maximum values. Likewise, in both study areas, the fungal aerosol concentration is greater in the morning than in the afternoon. Meanwhile, the sampling points in each library had generally tight distributions. The concentration through the campaigns is homogeneous, although the Banco de la República library is more variable than the CAJAMAG library. Despite this, the CAJAMAG library shows more peak concentrations, 90% of the time, and the concentration is below 90 CFU/m^3^.

**Figure 4:**
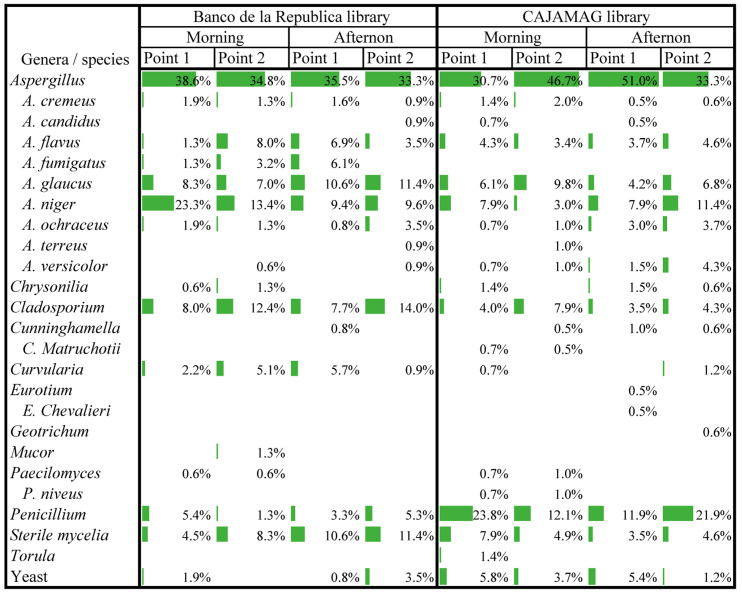
Percentage distribution of genera/species of fungi identified by library, the time of the day, and sampling point.

Figure 4 shows the eleven fungal genera that were identified (only eight of these in the Banco de la República library and ten in the CAJAMAG library). Moreover, nine species of *Aspergillus* and one of *Cunninghamella*, *Eurotium*, and *Paecilomyces* were identified. *Aspergillus* is the genera most abundant in both libraries, followed by *Cladosporium* and Sterile mycelia in the Banco de la República library; and *Penicillium* and *Cladosporium* in the CAJAMAG library. Among all the identified species, *Aspergillus niger* (*A. niger*) is the most abundant. Finally, the most frequent genus/species is *A. niger*, followed by *A. glaucus* and Sterile mycelia, which is congruent with the genera/species with higher concentrations.

### 3.2. Thermo-Hygrometric Conditions

**Figure 5:**
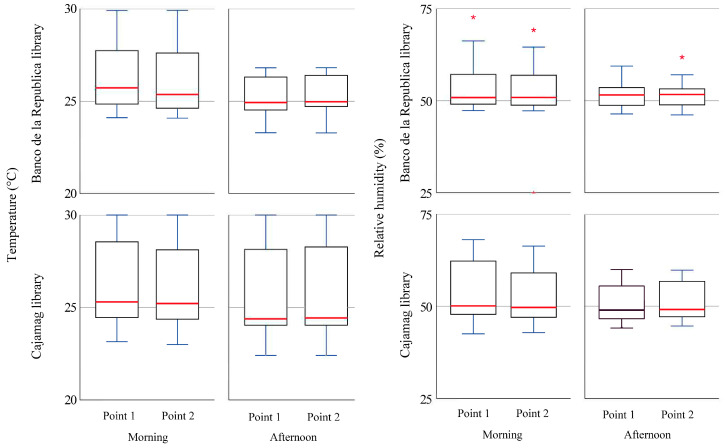
Thermo-hygrometric conditions in libraries. The left side is temperature. The right side is relative humidity. At the top is the Banco de la República library, and at the bottom is the CAJAMAG library. * Extreme value is 1.5 times more than the 75th percentile.

In the case of the thermo-hygrometric conditions, the results show 25.2 °C as the mean temperature and 52.1% as the mean RH. The temperature was homogeneous in both libraries, but the CAJAMAG library shows a slightly larger difference between the monitoring points, with higher temperature records in the morning than in the afternoon. The temperature varies more in the morning than in the afternoon, but to a greater degree in the Banco de la República library. In the case of RH, the values were more stable (with few changes) in the Banco de la República library and more varied in the CAJAMAG library. The behavior of the thermo-hygrometric conditions allows us to infer that the air mass in the libraries is generally homogeneous, with slightly more variability in the CAJAMAG library (Figure 5).

### 3.3. Statistical Results

**Figure 6:**
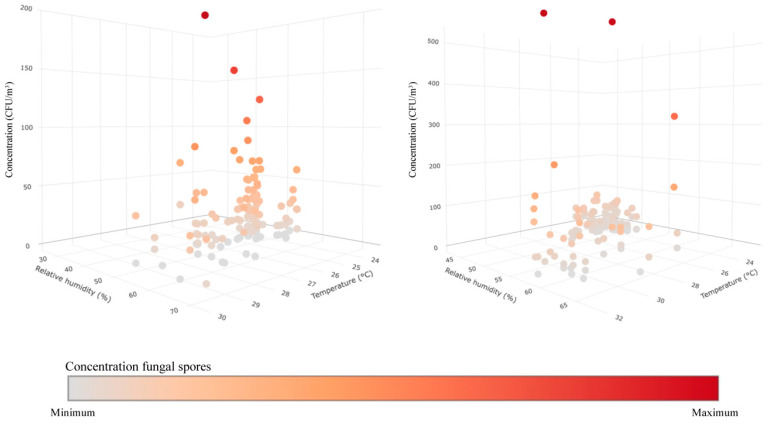
Correlation between thermo-hygrometric condition and airborne fungal spores’ concentration.

The result of the statistical test is displayed in Table 1. The thermo-hygrometric conditions only show a slight influence in the CAJAMAG library, where RH strongly influences the airborne fungal spore concentration. However, the correlation in all cases is weak due to the R^2^ coefficient explaining only 14% of the observed variance, despite the high statistical significance of 95%. This can be corroborated in Figure 6, which shows the graphical correlation between the variables.

### 3.4. Biodeterioration Risk Assessment Results

Table 2 shows the results of the potential risk of biodeterioration associated with airborne fungal spore concentration in the libraries. In general, the potential risk is medium in both libraries. However, the afternoon has slightly higher values than the morning. The difference could be explained by the fact that morning has more diversity of fungi aerosols but a lower concentration than the afternoon, which shows less diversity but a high concentration. Of the fourteen genera/species identified, *Aspergillus* is the genus that is recognized as having a high potential for biodegradation according to the literature, where *A. niger* is mostly reported as having high cellulose activity.

## 4. Discussion

Biodeterioration is defined as a negative change in the properties of paper materials, wood, textiles, and other items of cultural heritage by the action of microorganisms, and it produces significant damage worldwide. Some fungi have the potential to destroy cellulose and lignin and disperse spores to extend their range [17]. The biological activity of biodeterioration is influenced by environmental factors, as has been mentioned. The temperature (fungi can grow between 10 and 40 °C), humidity (some species of fungi can survive between 60 and 80%), dust (supplies a nutritive layer for the fungi and the nucleus for the moisture), pH (fungi grow in acidic conditions between 5.5 and 6.0, except *A. niger*, which grows in pH 2), and atmospheric pollution [17] have all been seen to impact the concentrations and species present.

In addition to environmental factors, the concentration of fungi allows us to infer the risk or potential state of biodeterioration of the material. In the present study, the reported values are low, compared with another investigation summarized by Camargo Caicedo et al. [13], except for the research conducted by Savković et al. [51], who reported a concentration of over 25,000 CFU/m^3^ in a closed cultural heritage conservation facility. In this work, the documents were older and possibly not always stored in the best conditions.

If we compare it with another study in the same city, the values are again relatively low; the difference is attributed to the ventilation condition. The present results correspond to closed libraries with artificial ventilation, while the other studies utilized natural ventilation [13]. This is consistent within the study area; the Caribbean aerobiological studies suggest high fungal spore concentrations associated with a high temperature and RH [52,53,54,55]; such conditions should influence the concentration of the library with natural ventilation [3,9,56] to a greater extent than artificially ventilated ones. However, it is of note that for 90% of the time, the concentrations are below 90 CFU/m^3^, which implies a low or null risk of biodeterioration. This is consistent with Rodríguez et al. [10], who posited that concentrations above 100 CFU/m^3^ are indicative of risk to library collections.

Climate conditions can encourage the growth and adaptation of fungi, and in a suitable habitat, this can lead to the secretion of various active metabolites, such as enzymes and acids, as has been reported in previous works [17,20,57]. In addition, the efficacy of isolated fungal strains in producing a variety of enzymes is characterized by their ability to secrete acidic metabolites, as was reported previously [18]. In our case, the climate conditions in the libraries show stable values throughout the day, and this explains the low correlation values and significance between thermo-hygrometric conditions and airborne fungal spore concentration; at the same time, this could be evidence that indoor environmental conditions are good for collection conservation. This agrees with Ruga et al. [14], who state that low variability of temperature and RH values in indoor conditions could reduce the airborne fungal spore concentration. Equally, Gallo et al. [58] argue that air conditioning systems can regulate the thermo-hygrometric variables, so if such parameters are adequately controlled, there is no risk of biodeterioration.

Statistical investigations have revealed that the highest percentages of infection in libraries are due to *Aspergillus* (approximately 30%) and *Penicillium* (more than 30%), and that the parts of the books that are most attacked are the first and last pages, the outer margins, and the binding [58]. Likewise, previous studies carried out in the repositories of the National Archive of the Republic of Cuba [2] coincide with the main genera identified in the present study: *Aspergillus*, *Cladosporium*, and *Penicillium.* In addition, the species *A. flavus*, *A. niger*, *A. terreus*, *A. versicolor*, *C. cladosporoides*, *C. elatum*, *C. fulvum*, *C. oxysporum*, *P. aurantiogroseum*, *P. commune*, *P. griseofulvum*, and *Mucor* are known to have qualitative cellulosic activity, produce organic acids, and excrete pigments onto paper [2].

Another study carried out on paper documents from the 19th and 20th centuries stored in the National Archive of the Republic of Cuba and in Argentinean archives reported the predominant genera *Aspergillus* (*A. flavus* and *A. niger* species), *Cladosporium*, *Fusarium*, and *Scopulariopsis*, and in lower concentrations *Eurotium*, *Paecilomyces*, and *Penicillium* [57]. Again, this is consistent with the results of the present study, and with the genera that have been reported previously in other studies [15,59,60]. Also, the predominance of *Aspergillus* in various climatic zones is explained by the cosmopolitan nature of this genus, as reported in the literature [26].

In our case, the most frequent species of *Aspergillus* is *A. Niger*, which was the most reported in the biodeterioration of paper due to its high cellulolytic activity. It reached a concentration of over 100 CFU/m^3^ several times during the sampling campaign. This could be of concern and be grounds for a higher biodeterioration risk value. However, the overall assessment risk was calculated to be medium for both libraries. This highlights the importance of knowing the precise airborne fungal spore concentration in an environment with artificial ventilation (closed indoor environment) and that the sources of microorganisms are relevant for indoor environments. Thus, if we recognize that the bibliographic material is a suitable substrate for the growth of fungi, their airborne fungal spore concentration can indirectly measure their presence in the books and, therefore, validate the establishment of the risk from their measurements. In that sense, aerobiological sampling would turn out to be more practical than the development of surface microbiological monitoring, especially in libraries with an extensive collection. This premise is corroborated by Ruga et al. [14], who monitored with two techniques the presence of airborne fungal spores’ concentration in a gallery in Italy with artificial ventilation, and assessed the behavior of the concentrations at different levels and rooms. They reported that rooms with similar structures, influenced by the same air masses, showed a correlation in fungal concentrations. Conversely, rooms that were not similar in this regard had a statistically different fungal concentration.

The biodeterioration of paper in books and documents depends on the enzymatic activity of microorganisms, so the intensity of fungi growth and the resultant damage is established by the fiber content in the paper and the type of glue used. The appearance of fungi in bibliographic material is associated with cellulolytic enzymes. Therefore, observations made by Strzelczyk and Leznicka [59] indicate that fungi are characterized by the active degradation of cellulose (i.e., the appearance of red stains on the edges and surface of the paper caused by the growth of *Penicillium*), as well as the formation of viscous substances.

No study is without its limitations, and this study is no different. Hence, there is a need to evaluate the methodology proposed here and to contrast the concentrations of the taxa identified in the bibliographic material and that of the airborne concentrations. This would greatly aid in precision, in terms of understanding the risk of biodeterioration. Future studies should include data on library use/users, activities, or mechanical movements that, in addition to the thermo-hygrometric conditions, potentially contribute to the release of fungal spores to evaluate the calculated risks.

## 5. Conclusions

The assessment of the biodeterioration risk caused by airborne fungal spores in two public libraries in the District of Santa Marta revealed that the CAJAMG library had the highest concentrations of fungi, reaching up to 300 CFU/m^3^. The average thermo-hygrometric conditions in both libraries are similar (25 °C, 50% RH), as well as the variability of the identified fungal genera. The most abundant species is *Aspergillus niger*, which is concerning due to its high potential for paper biodeterioration, posing a significant risk to the collections. Under the indicated environmental conditions, the potential risk of biodeterioration associated with the airborne fungal spore concentration in both libraries is medium.

## Figures and Tables

**Table 1:** Statistical regression results regarding airborne fungal spores in the libraries.

Library	Parameter	Variables in the Model		
		Temperature	Relative Humidity	All
Banco de la República	Equation	$y={\left(5.9-2.8\cdot 10^{2}x_{1}^{2}\right)}^{2}$	$y={\left(3.0+\frac{54.21}{x_{2}}\right)}^{2}$	$y=35.18-0.34x_{1}-2.78\cdot 10^{-2}\;x_{2}$
	R^2^	0.67%	0.30%	0.04%
	*p*-Value	0.319	0.503	0.950
CAJAMAG	Equation	$y=-69.8+0.15x_{1}^{2}$	$y=-62.5+3.6{\cdot 10}^{-2}x_{2}^{2}$	$y=-181.28+2.73x_{1}+2.91x_{2}$
	R^2^	12.59%	14.51%	14.13%
	*p*-Value	0.000	0.002	0.000

y: Concentration of fungal spore, $x_{1}$: temperature, $x_{2}$: relative humidity. P-critic: 0.005, if the *p*-value < P-critic, there is statistical significance with the 95% level.

**Table 2:** Potential risk assessment of biodeterioration per library and genera of airborne fungal spores.

		Banco de la República Library						CAJAMAG Library					
		Morning			Afternoon			Morning			Afternoon		
Genera/Specie	BP	Freq	CT	Risk	Freq	CT	Risk	Freq	CT	Risk	Freq	CT	Risk
*Aspergillus*	5	5	5	50	5	5	50	5	5	50	5	5	50
*A. cremeus*	5	2	2	20	2	2	20	4	4	40	1	1	10
*A. candidus*	1			0	1	1	2	1	1	2	1	1	2
*A. flavus*	5	4	4	40	4	5	45	4	5	45	4	5	45
*A. fumigatus*	5	4	4	40	4	4	40			0			0
*A. glaucus*	4	5	4	36	5	4	36	5	5	40	5	5	40
*A. niger*	5	5	5	50	5	5	50	4	5	45	5	5	50
*A. ochraceus*	5	2	2	20	2	4	30	2	2	20	4	5	45
*A. terreus*	5			0	1	1	10	1	2	15			0
*A. versicolor*	4	1	1	8	1	1	8	2	2	16	4	4	32
*Chrysonilia*	1	2	2	4			0	1	2	3	2	2	4
*Cladosporium*	4	5	5	40	5	5	40	4	5	36	4	5	36
*Cunninghamella*	4	1	1	8			0	1	1	8	1	1	8
*C. matruchotii*	4			0			0	1	1	8			0
*Curvularia*	2	4	5	18	4	4	16	1	1	4	1	2	6
*Eurotium*	4			0			0			0	1	1	8
*E. chevalieri*	4			0			0			0	1	1	8
*Geotrichum*	2			0			0			0	1	1	4
*Mucor*	2	1	2	6			0			0			0
*Paecilomyces*	4	1	1	8			0			0			0
*P. niveus*	4			0			0	2	2	16			0
*Penicillium*	4	4	1	20	4		0	5	5	40	5	5	40
*Sterile mycelia*	1	4	4	8	5	5	10	5	5	10	4	5	9
*Torula*	2			0			0	1	2	6			0
*Yeast*	1	2	2	4	2	4	5	5	5	10	4	4	8
Global risk		Medium (22)			Medium (26)			Medium (19)			Medium (23)		

BP: biodeterioration potential. Freq: frequency. CT: concentration thresholds. Red shading: very high risk. Orange shading: high risk. Green shading: medium risk. Blue shading: low risk.

## Data Availability

Data available on request.

## Supplementary Materials

The following supporting information can be downloaded at: https://www.mdpi.com/article/10.3390/jof10100680/s1, Figure S1. Cascade impactor viable diagram, its relationship to aerodynamic particle size and the human respiratory system; Figure S2. The protocol for quality assurance and control in samples.
