# Supplementary material for: Biodeterioration Risk Assessment in Libraries by Airborne Fungal Spores
Source: J Fungi (Basel). 2024 Sep 29;10(10):680. doi: 10.3390/jof10100680 (PMC11508409; doi:10.3390/jof10100680)
Supplement: Supplementary file 1 [file jof-10-00680-s001.zip › jof-3222213-supplementary.pdf]

Journal of *Fungi*

MDPI

Supplementary Material

Biodeterioration Risk Assessment in Libraries by Airborne Fungal Spores

Yiniva Camargo-Caicedo [1,2], Hilary Borja Pérez [2], Maryann Muñoz Fuentes [2], Eliana Vergara-Vásquez [1,2,*] and Andrés M. Vélez-Pereira [3,*]

1 Programa de Ingeniería Ambiental y Sanitaria, Facultad de Ingeniería, Universidad del Magdalena, Santa Marta 470004, Colombia; ycamargo@unimagdalena.edu.co
2 Grupo de Investigación en Modelación de Sistemas Ambientales-GIMSA, Facultad de Ingeniería, Universidad del Magdalena, Santa Marta 470004, Colombia; hilaryborja27@gmail.com (H.B.P.); marymunozfuentes@gmail.com (M.M.F.)
3 Departamento de Ingeniería Mecánica, Facultad de Ingeniería, Universidad de Tarapacá, Arica 1000000, Chile
* Correspondence: evergarav@unimagdalena.edu.co (E.V.-V.); avelezp@academicos.uta.cl (A.M.V.-P.); Tel.: +57-302-420-3779 (E.V.-V.); +56-58-220-7338 (A.M.V.-P.)

Citation: Camargo Caicedo, Y.; Borja Pérez, H.; Muñoz Fuentes, M.; Vergara-Vásquez, E.; Vélez-Pereira, A.M. Biodeterioration Risk Assessment in Libraries by Airborne Fungal Spores. *J. Fungi* **2024**, *10*, 680. https://doi.org/10.3390/jof10100680

Academic Editor: David S. Perlin

Received: 7 September 2024
Revised: 25 September 2024
Accepted: 27 September 2024
Published: 29 September 2024

Copyright: © 2024 by the authors. Licensee MDPI, Basel, Switzerland. This article is an open access article distributed under the terms and conditions of the Creative Commons Attribution (CC BY) license (https://creativecommons.org/licenses/by/4.0/).

1. Methodological support figures

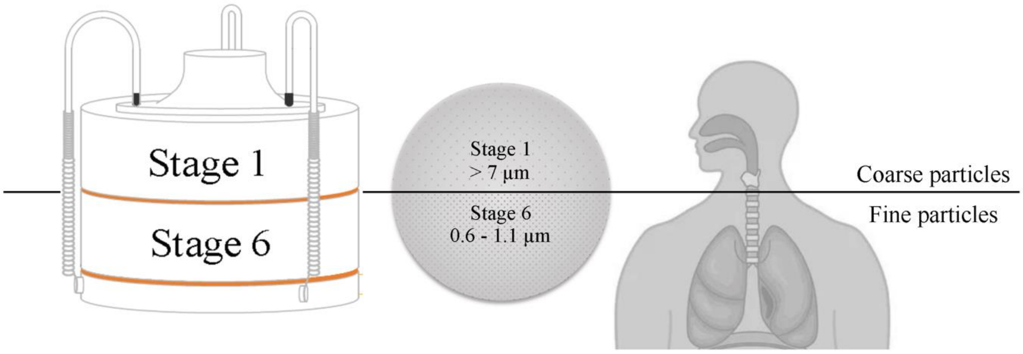

Figure S1. Cascade impactor viable diagram, its relationship to aerodynamic particle size and the human respiratory system. [1].

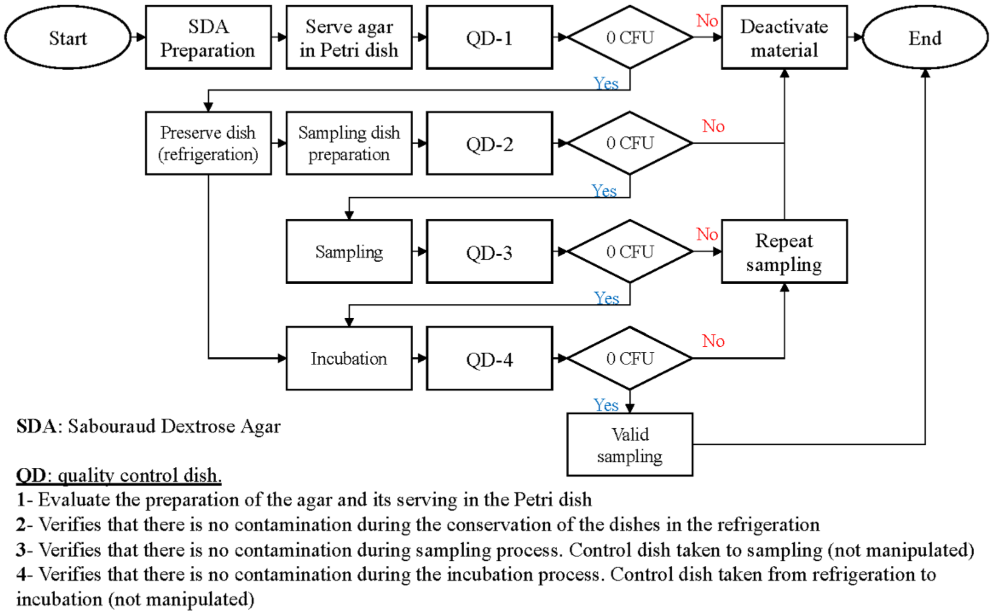

Figure S2. The protocol for quality assurance and control in samples. [1].

References

1. Camargo Caicedo, Y.; Borja Pérez, H.; Muñoz Fuentes, M.; Vergara-Vásquez, E.; Vélez-Pereira, A.M. Assessment of Fungal Aerosols in a Public Library with Natural Ventilation. *Aerobiologia* **2023**, *39*, 37–50, doi:10.1007/s10453-022-09772-5.

Disclaimer/Publisher's Note: The statements, opinions and data contained in all publications are solely those of the individual author(s) and contributor(s) and not of MDPI and/or the editor(s). MDPI and/or the editor(s) disclaim responsibility for any injury to people or property resulting from any ideas, methods, instructions or products referred to in the content.
